# Clinical, radiological profile and prognostic role of transcranial color-coded duplex in cerebral venous thrombosis: a case-control study

**DOI:** 10.1186/s12883-023-03342-z

**Published:** 2023-08-07

**Authors:** Alshaimaa M. Aboul fotouh, Sadek Mohamed Helmy, Husam S. Mourad, Hadeel Ahmed Abdelbaky, Ghada Hatem

**Affiliations:** Department of Neurology, Faculty of medicine, Cairo University, Al-Saraya Street, Al Manial, Cairo, Egypt

**Keywords:** Cerebral venous thrombosis, Hereditary thrombophilia, Transcranial color-coded duplex, Venous velocities

## Abstract

**Background:**

Cerebral venous thrombosis is a rare type of stroke, occurring more among young individuals. The presentation is highly variable, and this can delay diagnosis and management, thereby affecting outcome. The aim is to study the clinical, radiological profile, risk factors for cerebral venous thrombosis (CVT) and the role of transcranial color-coded duplex (TCCD) in CVT prognosis among Egyptian patients.

**Methods:**

Eighty CVT patients and 80 normal healthy individuals were included. Magnetic resonance imaging, magnetic resonance venography, and genetic thrombophilia tests were done for patients. Deep cerebral venous system was evaluated using B-mode transcranial color-coded duplex (TCCD) for both groups.

**Results:**

Showed female predominance with gender specific risk factors being the most common etiology. The most common hereditary thrombophilia was homozygous factor V Leiden mutation and anti-thrombin III (AT III). Headache was the most common presentation. Forty-three patients had transverse sinus thrombosis. Regarding TCCD, there was an increase in mean blood flow velocities, peak flow velocities and end diastolic flow velocities in deep middle cerebral vein and basal veins in CVT group compared to control group. There was a positive correlation not reaching statistical significance between flow velocities in the deep venous system and modified Rankin Scale.

**Conclusion:**

Clinical presentation is extremely variable. In our population, homozygous factor V Leiden mutation and AT III deficiency were the most common. Increased deep cerebral venous system flow velocities using TCCD in patients with CVT reflect their venous hemodynamic state.

## Background

Cerebral venous thrombosis (CVT) is a rare cause of stroke but is a serious disorder especially affecting individuals in their youth. The diagnosis can be missed because of myriad presentation mimicking other diseases [1]. The peak of incidence of CVT is in the third decade, with females more commonly affected than males with ratio 5:1.5 [2].

Cerebral venous thrombosis (CVT) is associated with multiple risk factors. Predisposing factors can be recognized in about 80% of cases [3]. Common predisposing factors are pregnancy, postpartum period, oral contraceptive pills, malignancies, hypercoagulable and inflammatory disorders, hematological disorders (as polycythemia and thrombocythemia) infections, dehydration, and head injury [4]. A person’s genetic background affect the risk of CVT where the risk is elevated when certain prothrombotic conditions are present, as factor V Leiden mutation, G20210A prothrombin gene mutation, methylenetetrahydrofolate reductase (MTHFR) gene mutation [5].

Contrary to arterial stroke, CVT may manifest as subacute (2–30 days) or chronic (> 30 days). The most common presentation of CVT is headache present in 88-93%, seizure in 37-71% and focal neurological deficit in 20-54% patients [5].

Currently, the diagnosis of CVT mostly depends on computed tomography venography (CTV) or magnetic resonance venography (MRV), but it cannot reveal the hemodynamic changes in deep cerebral veins, besides they cannot be used repeatedly for monitoring. Transcranial color-coded duplex (TCCD) is a non-invasive technique for measuring blood flow velocity in deep cerebral veins, such as the deep middle cerebral veins (dMCV) and basal veins (BV).

Additionally, it might indirectly reveal alterations in blood flow of the cerebral venous sinus, thus contributing to the monitoring of CVT [6].

## Methods

The aim is to study the clinical, radiological profile, risk factors for cerebral venous thrombosis (CVT) and the role of transcranial color-coded duplex (TCCD) in CVT prognosis among Egyptian patients.

This is a case control study conducted at Kasr Alainy, Cairo University Hospitals between May 2020 and January 2022. Included in this study 80 patients of both sexes above 16 years presenting with clinically and radiologically diagnosed CVT based on magnetic resonance venography (MRV) and patients who were two months not on anticoagulant two months before being recruited and 80 age and sex matched subjects.

This study was approved by the research ethics committee, Faculty of Medicine, Cairo University (code:MD-177-2020) and all patients provided informed written consent.

Excluded from the study individuals with CVT caused by local factors such as neurosurgical procedures, trauma, para infectious CVT, or occurred from a direct compression by a mass lesion (i.e., tumor), patients currently on anticoagulation for any reason, patients with incomplete or unusable medical records and patients with suspected cerebral venous thrombosis that was not verified by imaging.

Participants were subjected to thorough clinical assessment: including detailed history taking, complete general, neurological examination and modified Rankin Scale (mRS) after 3 months of CVT onset.

Protein C, S and antithrombin III (AT III) were done on all patients in this study while factor V Leiden mutation, MTHFR mutation, prothrombin G20210A and tests for antiphospholipid syndrome were done on patients without known risk factor.

Computed tomography of the brain (CT), magnetic resonance imaging (MRI) of brain (T1, T2 and FLAIR), magnetic resonance venography (MRV) was performed to all patients.

Transcranial color-coded duplex (TCCD) for cerebral venous system was performed at the neurosonology unit, of the Neurology department, Cairo University hospitals. It was performed by a single experienced certified neurosonographer using a high-resolution ultrasonography instrument (PHILIPS IU22 xMATRIX, California, US, L 1–5 transducer) equipped with a 2.5 MHz Phased array transducer.

Examination usually starts by insonation through the temporal acoustic bone window. The main segments of the circle of Willis serve as landmarks. The deep middle cerebral vein (dMCV) is found in proximity of the middle cerebral artery mainstem, and basal vein of rosenthal (BV) is found slightly cranial to the P2 segment of the posterior cerebral artery.

## Statistical methods

Data were coded and entered using the statistical package for the social sciences (SPSS) version 28 (IBM Corp., Armonk, NY, USA). Data was summarized using mean, standard deviation, minimum and maximum for quantitative variables and frequencies (number of cases) and relative frequencies (percentages) for categorical variables. Comparisons between groups were done using unpaired t test. For comparing categorical data, Chi square (χ2) test was performed. P-values less than 0.05 were considered statistically significant.

## Results

### Clinical parameters

Eighty patients were included; 63 patients (78.8%) were females, and 17 patients (21.3%) were males with age of disease onset ranging from 16 to 67 years with mean age 30.53 ± 8.66 years, summarized in Table (1).

#### Risk factors

Fifty three patients had identifiable precipitating factors subcategorized as follows: 23 (28.75%) had gender specific risk factors (8 (10%) had history of oral contraception use, 4 (5%) had history of abortion just prior to the onset,6 (7.5%) presented with CVT in postpartum period 2 of which had previous abortion, 5 (6.25%) patients were pregnant one of them with history of previous abortions), 13 (16.25%) had systemic lupus erythematosus (SLE) ,7 (8.75%) had history of previous deep venous thrombosis (DVT), 6 (7.5%) patients have Bechet disease and one patient (1.25%) with each of the following: overweight, recurrent DVT, Antiphospholipid syndrome and cancer breast, while no identifiable precipitating factors were found in the remaining 27 patients, summarized in Table (1).

**Table 1:** Clinical parameters and risk factors among the patient group

**Clinical parameters**	**Number**	**Percentage**
Females	63	78.8%
Males	17	21.3%
**Age at onset**	**Range**	**Mean ± SD**
	16–67 years	30.53 ± 8.66
**Risk factors**	**Number**	**Percentage**
1- Gender specific risk factors: a) Oral contraception use b) History of abortion just prior to onset c) Post partum period - With history of previous abortions - Without history of previous abortions d) During pregnancy - With history of previous abortions - Without history of previous abortions	23 8 4 6 2 4 5 1 4	28.75% 10% 5% 7.5% 2.5% 5% 6.25% 1.25% 5%
2- Systemic lupus erythematosus (SLE)	13	16.25%
3- History of previous DVT	7	8.75%
4- Behcet disease	6	7.5%
5- Overweight	1	1.25%
6- Recurrent DVT	1	1.25%
7- Antiphospholipid syndrome	1	1.25%
8- Cancer breast	1	1.25%
9- No identifiable precipitating factors	27	33.75

#### Clinical presentation

Headache was the presenting symptom in 68 (85%) patients of those 36 (45%) presented with isolated headache ,32 (40%) had headache associated with neurological deficits, while 12 (15%) patients didn’t present with headache, 7 (8.75%) of them with seizures and 5 (6.25%) with focal neurological deficits, summarized in Table (2).

Papilledema was found in 65 (81.25%) patients and 15 (18.75%) had normal fundus examination.

**Table 2:** Clinical presentation of CVT patients

	Number of patients	Percentage
Headache a) Isolated b) Associated with motor affection c) Associated with sensory affection d) Associated with seizures e) Associated with diminution of vision f) Associated with blurring of vision	68 36 10 2 9 1 10	85% 45.0% 12.5% 2.5% 11.25 1.25% 12.5%
Weakness: Right Left	11 7 4	13.8% 8.8% 5%
Sensory: Right Left	5 1 4	6.25% 1.25% 5%
Blurring of vision	10	12.5%
Diminution of vision	1	1.25%
Diplopia	1	1.25%
Seizures	17	21.3%
Papilledema	64	80%
Disturbed conscious level	20	25%

### Laboratory findings

Complete blood count (CBC), thyroid profile and kidney functions were done for all patients and were all normal.

Nine patients (11.25%) had AT III deficiency, 4 (5%) had protein C deficiency and 4 (5%) had protein S deficiency.

From 27 patients with no identified risk factors who underwent factor V Leiden and MTHFR mutation, prothrombin G20210A, antiphospholipid and autoimmune profile tests, 8 patients (29.6%) showed positive homozygous factor V and 3 (11.1%) had positive heterozygous factor V, shown in table (3).

**Table 3:** Thrombophilia testing in CVT patients

Thrombophilia test	Number of patients	Percentage
Protein C deficiency	4/80	5%
Protein S deficiency	4/80	5%
Antithrombin III deficiency	9/80	11.25%
Factor V Leiden mutation ^a^		
a) Homozygous b) Heterozygous	8/27 3/27	29.6% 11.1%
MTHFR mutation ^a^	1/27	3.7%

^a^ Done for 27 patients with CVT who have no risk factor, MTHFR: methylenetetrahydrofolate reductase.

### Radiological findings

- Computed tomography (CT) of the brain, MRI and MRV findings are shown in Table (4).
- Transcranial color-coded duplex (TCCD): Velocities in deep middle cerebral veins (dMCV; right and left) and basal vein (BV; right and left) showed increased peak systolic (PSV), end diastolic (EDV) and mean flow velocities (MFV) compared to normal age & sex matched subjects as shown in Table (5).

**Table 4:** Radiological findings in CVT patients

	Number of patients	Percentage
**CT brain**		
1-Normal 2-Venous infarction 3-Multiple venous infarctions ^a^ 4- Hemorrhagic infarction^a^	54 25 1 1	67.5% 31.25% 1.25% 1.25%
**MRI brain**		
1-Normal 2-Venous infarction: Right parietal Left parietal Right temporal Left temporal Right occipital Left occipital 3-Multiple venous infarctions ^a^ 4- Hemorrhagic infarction ^a^	47 32 8 5 2 4 5 8 1 1	58.75% 40% 10% 6.25% 2.5% 5% 6.25% 10% 1.25% 1.25%
**MRV**		
1-Superior sagittal sinus 2-Transverse sinus: Right Left bilateral 3-Multiple sinuses	32 13 30 1 4	40% 16.25% 37.5% 1.25% 5%

^a^ Same patient has multiple and hemorrhagic infarctions.

**Table 5:** Transcranial sonography findings in CVT patients compared to healthy control

	**Patients**		Control		P value
	**Mean+/- SD**	Range	Mean+/- SD	Range	
**Basal vein:**					
Mean flow velocity (MFV)					
a) Right b) Left	20 +/-9.35 18.6 +/-8.03	(10–43) (9–33)	9.34+/-1.17 11.65+/-1.65	(8–12) (8–13)	< 0.001^a^
End diastolic velocity (EDV)					
a) Right b) Left	17.5 +/- 7.94 17.5 +/- 7.10	(10–38) (8–29)	6.89+/-0.75 10.55+/-1.08	(6–8) (9–12)	< 0.001^a^
Peak systolic velocity (PSV)					
a) Right b) Left	18.8 +/-10.92 19.9 +/-6.96	(8–48) (10–33)	9.9+/-1 12.91+/-1.28	(9–12) (11–15)	< 0.001^a^
**Deep middle cerebral vein**:					
Mean flow velocity (MFV)					
a) Right b) Left	18.89+/-11.42 18.56+/-4.56	(9–44) (12–26)	9.9+/-1 12.91+/-1.28	(9–12) (11–15)	< 0.001^a^
End diastolic velocity (EDV)					
a) Right b) Left	17.11+/9.59 20+/-5.79	(7–35) (9–27)	7.56+/-1.52 11.34+/-1.43	(6–10) (9–13)	< 0.001^a^
Peak systolic velocity (PSV)					
a) Right b) Left	18.89+/-11.42 18.56+/-4.56	(9–44) (12–26)	9.9+/-1 12.91+/-1.28	(9–12) (11–15)	< 0.001^a^

^a^ Statistically significant

### Prognosis

The Modified Rankin Scale (mRS) scale after 3 months showed that 70 (87.5%) patients totally improved with mRS = 0, 1 (1.25%) with residual weakness mRS = 1 and 9 (11.25%) with seizures and mRS = 2.

Correlation between venous duplex velocities and outcome using mRS and with poor prognostic factors including Glasgow Coma Scale (GCS), seizures at onset and multiple sinus affection, revealed positive non-significant correlation between mRS and dMCV velocities, negative correlation with seizures at onset but not reaching statistical significance, positive correlation not reaching statistical significance between GCS at onset and BV velocities, while regarding multiplicity of venous affection there was significant positive correlation with PSV, EDV, MFV of basal veins and with the MFV of deep middle cerebral veins, shown in table (6)

**Table 6:** Correlations of TCCD findings

	Modified Rankin scale (mRS)		Seizure at onset		Glasgow coma scale (GCS) at onset		Combined sinus involvement	
	Correlation coefficient	P value	Correlation coefficient	P value	Correlation coefficient	P value	Correlation coefficient	P value
**1-Deep middle cerebral vein**								
Right mean flow velocity	0.063	0.601	-0.070	0.560	0.133	0.264	0.267	0.024 ^a^
Left mean flow velocity	-0.088	0.463	0.112	0.349	-0.16	0.179	-0.229	0.054^a^
Right peak systolic velocity	0.19	0.11	-0.050	0.675	-0.016	0.892	0.059	0.622
Left peak systolic velocity	0.218	0.066	-0.129	0.281	0.031	0.794	0.101	0.397
Right end diastolic velocity	0.105	0.378	-0.165	0.167	0.089	0.456	0.27	0.022 ^a^
Left end diastolic velocity	0.093	0.435	-0.003	0.977	-0.039	0.748	-0.218	0.066
**2-Basal veins**								
Right mean flow velocity	-0.119	0.295	-0.099	0.384	0.267	0.017 ^a^	0.228	0.042^a^
Left mean flow velocity	-0.03	0.789	-0.007	0.953	-0.082	0.469	0.105	0.356
Right peak systolic velocity	-0.066	0.562	-0.117	0.300	0.133	0.239	0.263	0.019 ^a^
Left peak systolic velocity	0.067	0.555	-0.208	0.064	0.177	0.116	0.246	0.028 ^a^
Right end diastolic velocity	-0.055	0.631	-0.134	0.237	0.208	0.064	0.253	0.023^a^
Left end diastolic velocity	0.121	0.283	-0.164	0.146	0.083	0.465	0.289	0.009 ^a^

^a^ Statistically significant

## Discussion

The age of our patients was 30 years on average, this agrees with several studies [7–9].

Female predominance was in agreement with Souirti et al. [10], Zuurbier et al. [11], and Yii et al. [12], studies in which females were more affected than males this is supported by the fact that the most frequently identified risk factor in our study were gender specific risk factors this came in accordance with International Study on Cerebral Vein and Dural Sinus Thrombosis - ISCVT, Cerebral Venous Sinuses Thrombosis Study - VENOST, Foschi et al., 2021, Koopman, et al., 2009 and Coutinho et al., 2009 [13–16]. This may be because hormonal causes results in prothrombotic condition (Ferro et al., 2005) [17].

The second most common cause was SLE, Cardona-Portela study and Singh study, found that CVT may be the initial presentation of SLE [18, 19].

There is increasing evidence associating underlying thrombophilic disorders to CVT [20–22], so we studied the hereditary thrombophilias and found that AT III deficiency was found in 11.25% of the patients presenting with CVT this result conforms with Khealani et al. [23], who found that 7% of their patients had AT III deficiency, we found that 5% of patients have protein S and C deficiency which is consistent with Pai et al. [24], and Martinelli et al. [25], respectively.

Minuk et al. [26], assumed that protein C and S testing is highly sensitive and specific even if measured during acute venous thrombotic event, also, Kovacs et al. [27], found that levels of protein C & S measured immediately after acute venous thrombotic event were the same as levels done after 3 months in 98% and that only 2.2% had false positive results. Additionally, 40% of patients have factor V Leiden mutation, this agreed with previous studies [28, 29].

We found 1 patient with MTHFR mutation this agree with Cantu et al. [30], who found no association between MTHFR mutation and CVT and Romero et al. [31], who found that MTHFR mutation is not considered risk factor for CVT. Besides, no patient was found to have PG20210A mutation, this was matching with findings in Aguiar et al. [21], study, where the incidence of CVT and the PG20210A mutation were not statistically correlated and with Rahimi et al. [32], who didn’t find this mutation in his study.

Cerebral venous thrombosis is characterized by their clinicoradiological polymorphism. In this study, headache was the most common presenting symptom (85.1%), followed by consciousness disorder (25.0%), seizures (22.5%) and focal neurological deficits (21%) this was concordant with Coutinho et al., Napon et al., 2010, Wasay et al., and Pathak et al. [16, 33–35],

The mechanisms underlying headache in CVT are not well known but many proposed mechanisms include increased intracranial pressure, stretching of nerves in wall of sinuses, inflammation of sinus walls and subarachnoid hemorrhage [36].

Focal neurological deficits occur depending on the area affected [37]. Seizures may occur because of disturbed blood–brain barrier resulting in brain edema with normal cortical neurons [38].

**VENOST** study, Khealani et al., and Sassi et al., 2017 [13, 23, 39] agreed with this study that showed that about half of patients had normal brain imaging, also we found that non-hemorrhagic infarctions were more common than hemorrhagic infarctions and this agreed with Walecki et al. [40], and Ferro et al. [38], but came contradictory to Naveen et al. [41], and Vidyasagar et al. [37], who stated that, hemorrhagic infarction was more common than non-hemorrhagic infarction.

In our patients transverse sinus (55%) was the most commonly involved sinus, followed by the superior sagittal sinus (40%) these findings are generally consistent with what has been previously reported in Deme et al. [42], study, Bousser and Ferro . [43], and ISCVT [38] which showed that the most affected sinus is transverse sinus (54.3%), followed by the superior sagittal sinus (38.6%), but this was contradictory to Zuurbier et al. [11], and Vidyasagar et al. [37], who found that superior sagittal sinus (80%) was most commonly involved followed by transverse sinus (64.4%).

Regarding the prognosis we found that 87.5% of patients had complete clinical recovery. These findings were in accordance with many studies in which complete clinical recovery (mRS = 0) was commonly reported in CVT patients [14, 44].

Assessment of cerebral venous hemodynamics by TCCD couldn’t be detected by other imaging techniques, so it can be used as a complementary imaging [45], also increased velocities represent an indirect sign of cerebral venous stasis [46], accordingly we found that velocities of deep middle cerebral veins and basal veins were higher in patients with evidence of sinuses thrombosis compared to healthy individuals. Valdueza et al. [47], found that blood flow velocities in deep cerebral venous system were higher in CVT patients and with recanalization of the venous system they revert to normal.

Decline in venous flow velocities in deep cerebral vein occurs due to either recanalization of occluded vein or formation of collateral circulation, resulting in neurological improvement [48]. We didn’t find that deep venous system velocities could predict outcome assessed by mRS despite the positive correlation with dMCV velocities that showed non-statistical significance, and this may be due to small sample size and that TCCD was not repeated to detect changes in velocities in relation to clinical symptoms. This agreed with Valdueza et al. [49].

Regarding poor prognostic factors, we found that deep cerebral venous flow velocities by TCCD in CVT patients was correlated with multiple venous system affection being higher in these patients reflecting the more severe venous stasis. There was negative correlation between deep venous system velocities and seizure but not reaching statistical significance, 88% of them have venous infarctions on MRI that occur because of the continued elevation in venous pressure resulting in cytotoxic edema and infarction [50], additionally brain edema exacerbates venous obstruction [51], causing a decrease in venous flow velocities.

Additionally, we found positive correlation between the blood flow velocities in the basal veins and GCS at onset though not of statistical significance this may be because basal vein of rosenthal act as a collateral, so their velocities are increased, with subsequent reduction in cerebral edema and accordingly improvement of consciousness [49] but in other study no correlation was found [45].

The limitations of this study were that TCCD was not repeated for patients and therefore we weren’t able to find out a significant prognostic value for TCCD. Some what the small sample size was another limitation.

## Conclusion

Clinical presentation is extremely variable, with headache being the most prevalent symptom.

Gender specific risk factors are the most common. Thorough exploration for an underlying systemic etiology in unprovoked CVT is useful for further long-term anticoagulation plan. TCCD evaluation of DCV blood flow is a quick, noninvasive, and secure method for identifying hemodynamic alterations in deep cerebral veins. It could help in predicting the prognosis, but this needs further studies and the use of contrast enhanced TCCD for better evaluation.

## Data Availability

All data generated or analyzed during this study are available from the corresponding author on reasonable request.

## Abbreviations

AT III: Anti-thrombin III
BV: Basal veins
CT: Computed tomography of the brain
CVT: Cerebral venous thrombosis
dMCV: Deep middle cerebral veins
DVT: Deep venous thrombosis
EDV: End diastolic velocity
GCS: Glasgow Coma Scale
MFV: Mean flow velocity
MRI: Magnetic resonant imaging of brain
mRS: Modified Rankin Scale
MRV: Magnetic resonance venography
MTHFR: Methylenetetrahydrofolate reductase
PSV: Peak systolic velocity
SLE: Systemic lupus erythematosus
TCCD: Transcranial color-coded duplex
